# Clinical Lectures on Diseases of the Eye

**Published:** 1864-02

**Authors:** E. L. Holmes

**Affiliations:** Lecturer on Diseases of the Eye and Ear in Rush Medical College, and Surgeon of the Chicago Charitable Eye and Ear Infirmary


### CLINICAL LECTURES ON DISEASES OF THE EYE.

By E. L. HOLMES, M. D., of Chicago,

*Lecturer on Diseases of the Eye and Ear in Rush Medical College, and Surgeon of the Chicago Charitable Eye and Ear Infirmary.*

### THE OPHTHALMOSCOPE.

Gentlemen: We have before us in the clinic to-day three patients, affected with various diseases, of which it is impossible to make an accurate diagnosis without the assistance of the ophthalmoscope. Before concluding what I wish to say upon diseases of the conjunctiva, I shall ask your attention to some of the principal points in the subject of ophthalmoscopie. You are aware there are two reasons, why it is impossible without the aid of some such instrument as the ophthalmoscope, to obtain a view of the deeper portions of the eye. 1st, On account of the smallness of the pupil, but little light can enter the eye. As nearly all of this is absorbed by the pigment of the choroid, a minute quantity of light only is reflected back through the pupil into the eye of the observer. The conditions are nearly the same in an attempt to look into a dark room through a small aperture in the shutter. Objects behind the iris, as also behind the shutter, are indistinct, or invisible, because they are insufficiently lighted. 2d. The refracting media of the eye so change the direction of the rays of light, as they pass from within out through the pupil, that they cannot meet at a focus on the retina of the observer. As the presence of a double convex lens in the aperture of the shutter, just mentioned as an illustration, increases the difficulty of looking into the room. Now if a piece of looking glass, from the centre of which a small disk of the amalgam has been removed, be placed near the opening in the shutter in such a way, that the light of the sun shall be reflected through this opening, the rays will fall upon the opposite wall. A person placing his eye behind the center of the reflector can plainly see those portions of the room upon which the light from the glass is thrown.

To throw sufficient light into the eye, so as to illuminate its internal structures and at the same time to so direct, by means of lenses, the rays as they pass back from those structures through the pupil, that they will form an image on the retina of the observer, are the objects of the Ophthalmoscope.

It consists of a small concave mirror with an opening in the centre and a semicircular frame behind it for holding such lenses as the peculiarities in the refracting media of the patient or observer may require. The instrument is used in the following manner: The room being darkened and a lamp with sufficiently strong light being brought near the patient's ear, the observer places the centre of the mirror directly before his own eye and inclines the mirror in such a way, that the rays of light from the lamp will be reflected into the pupil of the patient. It is usually necessary that the eye of the observer should be six to fourteen inches from that of the patient, the distance varying according as the patient or observer is far or near sighted. In this way an erect image of the retina can be obtained, so much magnified that but a small part of it can be seen at once. A slight movement of the instrument perpendicularly or horizontally will bring different portions of the retina into view. In order to examine a large portion of the retina at a glance it is necessary to use the mirror without a lens behind it, substituting a double convex lens of two or three inch focus, held between the mirror and the eye of the patient and very near the latter. It should be remembered that it is generally necessary to dilate the pupil of the patient by instilling a few drops of solution of neutral sulphate of atropine, gr. iv and aquæ ℥j, into the eye ten or fifteen minutes before making an examination. The study of the progress and effects of disease of the different tissues of the eye, as revealed by the opthalmoscope, is one of the most intensely interesting subjects in ophthalmic science. To acquire perfect facility in its use demands much experience and patient observation. But to enable yourselves to recognise diseases of the crystalline lens and vitreous humor, requires comparatively little labor. Even by means of a piece of common looking glass, used as I have just described in the illustration of the darkened room, you can with a little practice soon learn to detect opacities of the vitreous humor and of incipient cataract.

The ophthalmoscope is principally used in examining diseased conditions of the lens, vitreous humor, retina and choroid. It is occasionally employed in detecting differences of convexity in the different chambers of the cornia, as I shall show you in considering the accommodation of the eyes to different distances and its anomilies. It is well for you before attempting to use the ophthalmoscope to examine repeatedly by simple inspection many normal eyes of different individuals of different ages, in order to be accustomed to notice the condition of the cornea, its distance from the iris, the size and shape of the pupil, and the *color* of the lens, which last, you are aware, in advanced life is normally of a yellowish tint, not to be confounded with the discoloration of cataract. After being able to examine the eye well in this way, you should commence the examination with the ophthalmoscope of patients affected with complete amaurosis, in whom the transparent media are normal. In these cases, the patient will not become fatigued by long and repeated examinations. In this manner you will be able to recognize at a glance when the transparent portions of the eye are normal. The orange red disk which you will observe as you look through the pupil will serve as a guide in recognizing abnormal changes. For example, if you observe small dark spots in the disk situated in the plane of the pupil you may be certain they are situated on the anterior surface of the lens; if situated just back of the iris and near the circumference of the disk, especially if they radiate towards the centre, you may state confidently that the patient is afflicted with incipient cataract. If, as the patient rotates the eye, you observe dark irregular bodies moving in different directions, especially upwards and downwards, you know that there are floating masses of pigment in the vitreous humor. Sometimes these collections of pigment do not change their relative position, but are fixed in the vitreous humor. Sometimes you are able to detect recent hœmorrhages from the choroid, retina, and other tissues and foreign bodies in the vitreous humor.

I will here call your attention to another mode of examining the eye. If you stand facing your patient, placed in such a position, that the light from a window, or rather from a bright lamp, falling through a double convex lens of two and a half inch focus obliquely upon the eye, will become concentrated in the pupil, you will secure an excellent view of all that is situated in the pupil or just behind it. I advise you to become familiar with this mode of examining the eye, for it is very advantageous as at the same time simple. Thus far the examination of the eye with the ophthalmoscope through a dilated pupil is simple and requires comparatively little practice. The investigation of the abnormal conditions of the retina and choroid, however, is a much more difficult matter. The selection of the right lens and the adjustment of the ophthalmoscope at the suitable distance from the eye, to secure a distinct image, are points of difficulty not readily overcome by the beginner in the use of the instrument. As I have already stated, the most convenient cases for the beginner to examine, are those where there is complete amaurosis and dilatation of the pupil, since a strong light can be thrown into the eye for a long time without fatigue to the patient. The appearance of the retina as seen in the dissected eye has already been demonstrated to you. The fine vessels and the end of the optic nerve, where it enters the sclorotic, which is scarcely a half line in diameter, appear very much magnified through the ophthalmoscope. The former can be seen passing in nearly all directions, principally however, upwards and downwards from the centre of the optic nerve, which appears as a large round well defined disk of a very pale orange color. Some of the most important pathological changes in the retina which can be observed by the ophthalmoscope, are deposits of pigment, exudations of lymph, under and in the retina, detachments of the retina from the choroid, and obliteration of the vessels. Changes in the appearance of the optic nerve also, as regards size, form, and color, may be readily seen. In the choroid, absorption and increased deposition of pigment, extravasation of blood, and some of the products of inflammation can be seen with the ophthalmoscope as plainly as corresponding changes in the iris can be recognized by the naked eye.

I might mention other abnormal conditions, but those to which I have already alluded are sufficient to demonstrate to you how indispensable the ophthalmoscope is in forming an accurate diagnosis in diseases of the interior structures of the eye.

I should not omit to urge upon you care in the use of this instrument where there is photophobia or active inflammation of any of the tissues of the eye. When, however, the patient can bear to look directly at the light of an ordinary lamp held quite near the eye, there can be no danger in using the ophthalmoscope. As you gain experience in the use of the instrument, you will find not only that you can make an examination with greater rapidity, but also with a smaller amount of light. It is always your duty to inform patients that the dilatation of the pupil produced by Belladonna or Atropine, will cause for a few days an indistinctness of vision and a certain degree of photophobia, which may require a common shade over the eyes and perfect rest.

Nearly all the recent works upon diseases of the eye contain more or less upon Ophthalmoscopy. If you wish to comprehend the whole subject you must study the theory and use of the instrument, and also the pathology of the retina and choroid, as given in the Archive for Ophthalmology, in the works of Arlt, Stellwag, E. Jaeger, and Hogg. The beautiful colored plates of Jaeger and of Liebreich, which I show you here to-day, will give you an excellent idea of abnormal conditions as revealed by the Ophthalmoscope.

